# Posttraumatic Growth as a Pathway to Wellness for Individuals and Organizations

**DOI:** 10.3390/bs15121653

**Published:** 2025-12-02

**Authors:** Richard G. Tedeschi, Bret A. Moore, Taryn C. Greene

**Affiliations:** Boulder Crest Institute for Posttraumatic Growth, Bluemont, VA 20135, USA; bret.moore@bouldercrest.org (B.A.M.); taryn.greene@bouldercrest.org (T.C.G.)

**Keywords:** trauma, resilience, posttraumatic growth, organizational change, wellbeing

## Abstract

Posttraumatic growth (PTG) offers an alternative understanding of trauma response, contrasting with traditional perspectives focused solely on symptom development and resilience. In the PTG model, individuals and groups report positive changes in life philosophy, self-understanding, and interpersonal relationships because of successfully navigating the struggles involved with experiencing adversity. This narrative review includes the evolution of PTG theory, highlighting the disruption of core beliefs, the role of rumination, and the reconstruction of a life narrative as central mechanisms of the PTG process. The authors describe the five empirically validated domains of PTG and outline the naturally occurring PTG process. Methods are discussed for facilitating PTG through “expert companions,” who may be peers or professionals providing nonjudgmental, supportive relationships that encourage emotional regulation, constructive disclosure, and meaning making. The article explores how these methods can be applied to individuals, communities, and organizations, all of which may experience comparable domains of growth following collective adversity. The article concludes by highlighting how a focus on posttraumatic growth shifts perspectives from viewing trauma survivors as broken to recognizing their potential for transformation and underscores the role of both professional and community support in fostering PTG and healthier organizational climates.

## 1. Introduction

In the 1990s, the term *posttraumatic growth* was introduced to the psychological lexicon by Tedeschi and Calhoun (1995, 1996). Since then, there have been thousands of studies devoted to this topic. Although this is a relatively new concept in the psychological literature, the experience of posttraumatic growth has been described in other terms (e.g., Finkel, 1975; Taylor, 1983) in one form or another in the literature and religious texts over the millennia. In this article, we briefly describe the concept and examine how it applies to experiences of individuals who are struggling in the aftermath of trauma and also to organizations that are subjected to disruptive change. We refer to change processes involved as well as the particular outcomes that are often seen as a result of this process. We also distinguish it from the related concept of resilience.

Posttraumatic growth represents the positive changes that may occur as a result of the struggle in the aftermath of adverse or potentially traumatic events. This struggle is with the cognitive and emotional effects of traumatic events. Tedeschi and Calhoun built on the trauma response work of Herman (1992) and Janoff-Bulman (1992) in describing the ways events can create a challenge to the core belief system, or assumptive world, on which individuals’ basic understandings of the world are built. Core beliefs include foundational concepts of identity, sense of predictability and controllability, belief in justice and fairness, and concepts of morality. When events transpire that call into question or even shatter these basic assumptions, this constitutes trauma for the individual. These challenges to core beliefs generate confusion and anxiety, which leads to distressing rumination as individuals struggle to make sense of their circumstances and reconstruct their fundamental assumptions.

The metaphor of a natural disaster such as an earthquake has been used to describe the psychological effects of these challenges to the core beliefs or assumptive world (Tedeschi et al., 2026). This system of beliefs is like a psychological infrastructure that is akin to the infrastructure on which a city runs. When that infrastructure is shattered through trauma, it needs to be rebuilt, and it is best rebuilt to accommodate future disasters. Just as the people in the city must reconsider the configuration of city structures, individuals must reconsider and restructure their belief systems to make sense of what has happened and become more resilient in the face of future events. This is where we see the relationship between posttraumatic growth and resilience, since a successful process of posttraumatic growth can lead to an individual who is more resilient to future challenges. However, posttraumatic growth represents a transformative change in the individual rather than a resistance to change and adversity that defines resilience.

## 2. Posttraumatic Growth Process

The process of posttraumatic growth that begins with core belief disruption and can produce resilience and other important changes has been outlined in a number of different writings from 1995 to 2026, with several revisions reflecting the empirical literature on posttraumatic growth over this time (Tedeschi et al., 2026). There are several important phases of this posttraumatic growth process. Before examining these phases, it is important to first consider the unique characteristics of the person who has experienced traumatic change, as these will influence their ability to navigate the phases of the PTG process. There may be certain characteristics that allow individuals to adapt more readily to these challenging posttraumatic changes, including openness to experience and extraversion (Tedeschi & Calhoun, 1996). The former characteristic may allow individuals to more readily experiment with changes in their cognitive and emotional life, while the latter includes a greater tendency to connect with other people for support and understanding. Due to these individual factors, the PTG process unfolds differently for each person. Often, in the immediate aftermath of traumatic events, people experience periods of rumination characterized by intrusive thoughts, images, and emotions as they struggle to make sense of what happened to them. In the posttraumatic growth process, this intrusive rumination segues into a period of more deliberate rumination, which involves a more controlled and reflective process of rebuilding the core belief system. People can engage in this deliberate rumination as they develop ways to regulate their emotions, especially the anxiety that is part of this process. Emotional regulation and deliberate rumination can be enhanced through the development of interpersonal relationships that promote emotional support and new ways of thinking about the events that have occurred, contemplation of one’s value in the aftermath of these challenges, and the new life path that is necessary to traverse.

In the posttraumatic growth process, supportive others, who are called expert companions, can be crucial. These expert companions are people who encourage trauma survivors to explore the changes that they are embarking on with a consistent response of acceptance and encouragement (Tedeschi & Calhoun, 2009). In addition to the expert companions who provide an arena for self-disclosure and self-analysis, there may be more distal social and cultural influences that allow individuals to explore new ways of understanding what has happened and the direction of their life going forward. In the process of posttraumatic growth, this deliberate rumination can involve various kinds of analysis and disclosure as individuals revise cognitive schema and start to reconstruct a life narrative that recognizes the ways that adversity has been leading to important personal changes (Lindstrom et al., 2013). Over a period of time that is variable from individual to individual, there is an acceptance of this changed world and this changed self that leads to a variety of potential posttraumatic growth outcomes (Triplett et al., 2012).

## 3. Posttraumatic Growth Outcomes

Qualitative and quantitative work by Tedeschi and Calhoun (1996) led to a description of five different potentially positive outcomes in the aftermath of trauma. These five domains of posttraumatic growth are reflected in a measurement instrument called the Posttraumatic Growth Inventory (PTGI) whose factors have been substantially supported in a number of studies over various cultures (Tedeschi et al., 2017, 2026). One of the five domains reflects a pathway to resilience and is called personal strength. In this domain of posttraumatic growth, people recognize that they are stronger than they thought they might be or that they have been strengthened in some way by having to manage traumatic events. They are more confident in their ability to confront difficulties in the future because of the work they have done in rebuilding their core belief system. A second domain of posttraumatic growth involves relating to others. Individuals report a deeper understanding and more compassion and empathy in their relationships. These changes appear to occur because of their experiences with expert companions who demonstrate the way of relating that trauma survivors find so valuable. They have also experienced, out of necessity, a greater ability to disclose and therefore develop closer relationships. A third domain of posttraumatic growth is appreciation of life, which is apparent in a heightened sense of gratitude and newfound recognition of the value of experiences that have previously been overlooked. A fourth domain of posttraumatic growth is new possibilities. The events that confront individuals sometimes prevent them from continuing down paths they had previously been traveling and force them to consider different goals or priorities. In other cases, the process of posttraumatic growth has exposed them to individuals and circumstances that create opportunities that they might not have previously considered. The fifth domain of posttraumatic growth is spiritual and existential change. These are changes to foundational considerations of the meaning and purpose of life, and a sense of devotion to something greater than oneself. These changes can involve acts of service or commitment to principles and a sense of increased wisdom. It is important to note that these posttraumatic outcomes must be understood as a continuing process of personal growth and development rather than destinations. Therefore, the process of posttraumatic growth and associated outcomes are married into life experience that continues over time and contributes to a sense of change in the life narrative.

In the description of the process of posttraumatic growth and the five domains of outcomes, it is apparent that a variety of theoretical perspectives in psychology are represented (Tedeschi & Moore, 2021). Posttraumatic growth has a cognitive element, as it is built on the concept of core belief challenge and schema change. Posttraumatic growth also relies on the concept of narrative development as individuals create a new life script that has deviated from their assumptions about their plans and goals before the trauma occurred. It also emphasizes existential themes in the crucial role of meaning and purpose as people revise their life narratives (Tedeschi & Riffle, 2016). Consideration is given to the interpersonal process that encourages posttraumatic growth, and this relational element is critical in the posttraumatic growth-focused intervention process. Expert companions support trauma survivors through the cognitive and emotional challenges that follow trauma and help facilitate the discovery of new life paths, evolving priorities, and the emerging value of relationships.

## 4. Facilitation of Posttraumatic Growth

Posttraumatic growth often occurs naturally, but as we have stated above, supportive others have been shown to be an important factor in the posttraumatic growth process. Facilitation of this posttraumatic growth process can occur as expert companionship is available and accessed successfully by individuals as they navigate their challenging circumstances. It is important to understand that expert companions can be mental health professionals but are more likely to be lay persons who either serendipitously appear in trauma survivors’ lives or who are sought out as peer support who may provide understanding and guidance because they are familiar with the kind of circumstances that the individual confronts. Therefore, expert companions may be mental health professionals, or people who are available in the interpersonal environment as neighbors, friends, family members, coaches, teachers, clergy, etc. Or they may be peers who are found through support groups and nonprofit organizations (Rhodes et al., 2024).

No matter who they are, expert companions appear to have an ability to patiently listen without feeling compelled to direct or advise. They are non-judgmental as they recognize and accept that the struggle in the aftermath of trauma leads people to have difficulty with thinking clearly, controlling anxiety, and continuing to meet their obligations. Expert companions are interested in learning from the person who is experiencing trauma and its aftermath in order to understand that person’s particular circumstances. Because it is difficult to understand the experience of another who is struggling, peers who are trauma survivors themselves can be very effective in providing the kind of support that can lead to posttraumatic growth.

Some of the ways that mental health professionals are trained can interfere with this growth process rather than facilitate it. For example, they may become preoccupied with symptoms, diagnosis, and technical forms of intervention to address symptoms, which can be counterproductive. Mental health professionals need to be aware that what is most effective in their interventions involves their relational capacity, and there is good evidence in the psychotherapy outcome literature to support the critical importance of the therapy relationship (Norcross & Wampold, 2019). Therefore, in facilitating posttraumatic growth, mental health professionals must focus on their relationship rather than emphasize technical considerations such as desensitization procedures and other specific trauma interventions. These kinds of interventions can be useful but are best practiced within a relationship of expert companionship (Calhoun & Tedeschi, 2013).

Clinicians and non-professionals alike can follow the posttraumatic growth process in supporting trauma survivors by noticing five phases of the posttraumatic growth process that can unfold (Tedeschi & McNally, 2011). These phases are iterative and recursive and provide a framework that requires a constant focus on nurturing the interpersonal relationship with the survivor of trauma. In the clinical setting, this process can be integrated with other trauma-focused therapy practices, as there is overlap with them, given the integrative nature of the posttraumatic growth model.

*The Five Phases of Expert Companionship*. An important phase of the posttraumatic growth process is psychoeducation about the psychological and physiological effects of trauma but extended to a presentation of the importance of reconfiguring the core belief system in order to generate a new understanding of oneself and one’s life going forward. A second phase of this facilitation process involves providing some help in developing emotional regulation practices that are useful in reducing intrusive rumination and encouraging more deliberate rumination so that a revised and resilient core belief system may be constructed. A third element is facilitating constructive disclosure in an effort to gain more understanding about the state of the core belief system, and also to elicit information about the state of support the individual has access to. An important way one can support trauma survivors is to help them select expert companions in the social environment to connect with to nurture stronger and deeper interpersonal relationships. A fourth phase of this process is co-development of the personal narrative which integrates the before, during, and posttrauma experiences of the person into an overall understanding of the life course and the place of traumatic events in personal growth. A fifth phase of this process involves the development of a sense of meaning and purpose that can be focused on service and benefit to others. This turns the pain and suffering of traumatic events towards something meaningful that can be better endured. It should be apparent that cognitive, relational, narrative, existential, and behavioral elements of the therapeutic enterprise are all integrated in this process. An example of a case that utilizes this process can be found in Tedeschi (2011), and guidance on the process is found in Tedeschi and Moore (2016b).

## 5. Community and Organizational Posttraumatic Growth

The process by which posttraumatic growth occurs in individuals can also be observed in groups of individuals or organizations. Where groups of individuals are concerned, posttraumatic growth is examined as an aggregate. When considering organizational-level posttraumatic growth, the effect on individuals within the organization is considered, but also the structure of the organization in terms of its values and beliefs reflected in policies and actions.

Some examples of the impact of trauma and posttraumatic growth in groups of individuals include interventions with high school students (Taku et al., 2017), ex-offenders (Mapham & Hefferon, 2012) and military veterans (Kelley et al., 2025). Sometimes individual changes in posttraumatic growth can be observed at the community level as widespread improvement across a population. Kirkner and Ullman (2020) reported on a longitudinal study of posttraumatic growth predictors among a large number of sexual assault survivors in a community and reported that changes in core beliefs, perceived control over recovery, engagement with religious practices, positive social support, and fewer trauma symptoms predicted posttraumatic growth. Collective level posttraumatic growth was also reported by Wlodarczyk et al. (2016), where individual and collective posttraumatic growth were positively correlated and observed in the aftermath of natural disasters in Spain, Chile and Colombia. An example of collective posttraumatic growth that can be seen as an organizational change is the work of Williams et al. (1999), who reported on the response of the city of Kobe, Japan, to an earthquake in which 6000 people died. As a result of this experience, there was a new recognition of the importance of volunteers in such circumstances and that in the aftermath of such disasters, mental health services can be useful.

The COVID pandemic was a significant organizational challenge and can be considered an example of a traumatic event for organizations of all sizes. Organizations, like individuals, can also proceed with the process of posttraumatic growth and experience versions of the five domains. Tedeschi (2020) described how posttraumatic growth could be a basis for organizational change and development during the period of the COVID pandemic. Organizations can be thrown into chaos and struggles for survival because of external circumstances, as occurred during the COVID pandemic. This can set in motion a process of anxious reaction among people in the organization that also can lead to reflection and self-analysis akin to deliberate rumination. Organizations can find valuable expertise internally or through external consultation in order to set a new direction. A new business plan can act analogously to a narrative that has been revised. When we consider the five domains of posttraumatic growth at an organizational level, it is apparent that each domain has an organizational analog. Strength can be perceived as the organization navigates its difficulties and finds value in its employees. New possibilities can emerge in terms of processes or markets that have previously been unexplored. Relationships can be improved among employees and with clientele as the organization changes its perspectives and priorities. These new priorities can result in a newfound appreciation for the organization’s value in providing goods or services, and this can lead to a greater sense of purpose for those who work in these organizations and for the organization’s vision and mission.

Organizations that employ people often exposed to potentially traumatic circumstances have a particular responsibility for caring for their employees in understanding the process of trauma response. How the organization manages to support its employees is critical for employees’ mental health in these scenarios (Shakespeare-Finch et al., 2024). There are reports of some organizations of public safety personnel that have adopted trauma-informed care approaches to support their employees (Cherry et al., 2021; Creamer et al., 2012). Although such organizations may be providing structures and processes that benefit their employees who are exposed to potentially traumatic events, when people in the organizations understand these processes and responses, they may also be in a better position to apply these concepts to the organization as a whole as they navigate circumstances challenging for the organization (Scully, 2011). Whether it is a response to the needs of individual employees in order to support their mental health, or to the needs of the organization to ensure that it remains healthy in the face of challenges, people at all organizational levels play an important role in responding to these challenges (Kalmbach, 2024). People in leadership positions must align with those in direct service positions in designing and implementing policies and procedures to maintain the health of the organization and its employees in the face of challenge. Leaders can make use of the valuable experiences of their employees and include them in implementing programs that are meant to support them and the organization as a whole. Employees at the level of direct service need the support of their leadership as they operate as expert companions in positions of peer support, where they are trusted by their fellow employees.

## 6. Posttraumatic Growth-Based Perspectives and Policies

In approaching trauma as mental health professionals and in public policy, an emphasis on posttraumatic growth changes the perspective on trauma survivors from one of victimhood, brokenness, and disorder to one that highlights the possibility for transformation. This perspective changes how we relate to these individuals and also creates a focus in our conversations with them on highlighting their value, strengths, and continued purpose. There are indications that this sort of approach can at the same time have a significant effect on reducing PTSD symptoms (Kelley et al., 2025; Tedeschi & Moore, 2016a). The expert companion perspective that allows for the contributions of peer and community support persons in promoting posttraumatic growth suggests a democratization of the responsibility for maintaining mental health beyond the professional community (Patel et al., 2023). When people at all levels of organizations understand the posttraumatic growth concept and model, they gain the opportunity to create healthier organizational climates and greater responsiveness to the rapid changes and challenges that confront organizations at the individual level as well as the organizational level following trauma. This concept of posttraumatic growth can be understood as a recognition of humanity as a collective characterized by an inherent capacity for development and transformation in the face of all types of circumstances (Tedeschi et al., 2026). PTG emphasizes the innate human tendency not only to surmount adverse circumstances but to adapt, learn, and flourish in their aftermath, underscoring the importance of fostering environments and practices that more effectively support this fundamental human endeavor. In closing, the authors express the hope that this article equips people in all kinds of roles—from neighbor to co-worker to mental health professional—to intentionally encourage and facilitate growth in each other amidst the inevitable challenges and adversities that life presents.

## Data Availability

Not applicable.
